# Cerebral Abscess Potentially of Odontogenic Origin

**DOI:** 10.1155/2015/267625

**Published:** 2015-02-01

**Authors:** Marouene Ben Hadj Hassine, Lamia Oualha, Amine Derbel, Nabiha Douki

**Affiliations:** ^1^Department of Oral Medicine and Oral Surgery, Oral Health and Oro-Facial Rehabilitation Laboratory Research (LR12ES11), Faculty of Dental Medicine, University of Monastir, avenue Avicenne, 5019 Monastir, Tunisia; ^2^Department of Oral Medicine and Oral Surgery, Hopital Universitaire Farhat Hached, rue Ibn Jazzar, Ezzouhour, 4031 Sousse, Tunisia; ^3^Department of Conservative Dentistry and Endodontics, Hopital Universitaire Sahloul, Route de la Ceinture C. Sahloul, Hammam Sousse, 4011 Sousse, Tunisia

## Abstract

Odontogenic origins are rarely implicated in the formation of brain abscesses. The relative paucity of this kind of infection and the difficulty in matching the causative microorganisms of a brain abscess to an odontogenic source can explain the late management of patients. We herein describe a case of a 46-year-old man with a cerebellar abscess that was probably due to an odontogenic infection. The diagnosis supported by imaging and microscopic identification, mini craniectomy for abscess drainage followed by eradication of all potential dental infectious foci, and an antibiotic regimen based on cephalosporins, metronidazole, and vancomycine contributed to a successful outcome.

## 1. Introduction

Cerebral abscesses are rare but serious and life threatening infections and constitute a localized zone of suppuration within the brain. They usually follow cranial trauma or surgery or can be secondary to a septic focus elsewhere, spread either directly or haematologically [1]. The mortality rate was between 30 and 60% in the early 1970s but has decreased to 0 to 24% [2, 3] due to increased availability of neuroimaging techniques, such as computed tomography (CT) and magnetic resonance imaging (MRI), more effective antibiotics, and improved surgical techniques [4]. Rarely are odontogenic infections implicated in the formation of brain abscesses [5]. Owing to the low incidence of this infection, the challenge consists of recognizing it and preventing a delay in treatment, because the earlier the diagnosis is done, the higher the survival rate is and the lower the complications are seen [6, 7].

The aim of this paper is to show that early and adequate care helps prevent serious complications.

## 2. Case Presentation

A 46-year-old man, with no history of systemic disease, was complaining about sudden onset of balance disorders and dizziness for 10 days. These symptoms were marked by severe headaches and several episodes of vomiting. He also reported having undergone a tooth extraction one month ago which was complicated by fever according to his own statements. Therefore, he consulted a neurologist who diagnosed intracerebral hypertension and a cerebral scan was then ordered and revealed, indeed, the presence of 3 cerebellar ring-enhancing mass lesions of variable size measuring 28∗20 mm and 15∗20 mm. Afterwards, he was transferred to the Emergency Department (ED) at the University Hospital Center of Sahloul, Sousse, for further investigations.

On physical examination, he was found to have ataxic walking with no loss of feeling in the skin and muscle weakness.

With all these findings, he was admitted under the care of the neurosurgery service for a potentially intracerebral abscess. Given this information, the infectious diseases service was consulted to determine the most appropriate antibiotic treatment to begin. With that service's recommendation, the patient was started on metronidazole 500 mg intravenously every 8 hours, vancomycin 500 mg IV every 6 hours, and cefotaxime 2 g IV every 4 hours.

He was then taken to the operating room for a right retrosigmoid mini craniotomy for abscess drainage. Brain abscess specimens were submitted to microbiology, and the bacterial cultures returned positive exclusively for numerous cocci gram + (*Staphylococcus aureus*).

Besides, blood and urine samples were negative. Upon receiving these results and taking into account the fact that the patient underwent an extraction one month ago, the dentistry service was consulted to identify a possible odontogenic focus.

On extraoral examination, there was no cervical or submandibular lymphadenopathy.

**Figure 1:**
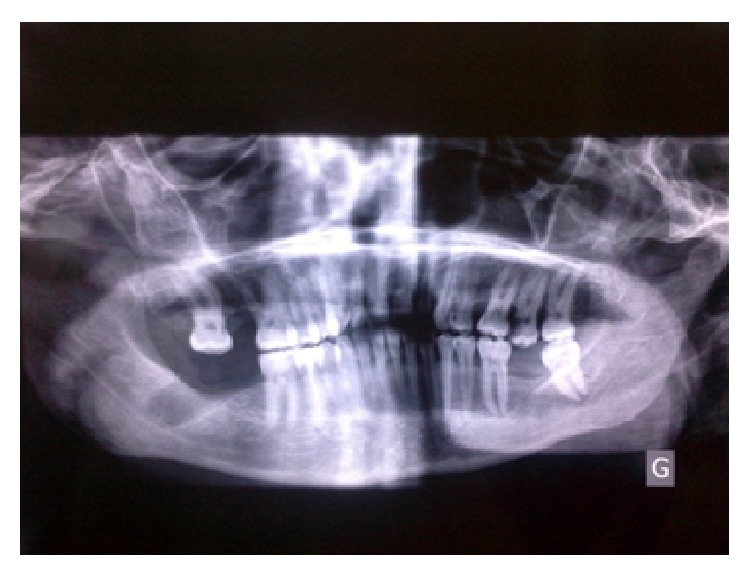
Preoperative panoramic radiograph.

There was no overt trismus or facial swelling. On intraoral examination, the patient was found to have a poor oral hygiene. The left maxillary second molar was grossly decayed, and the right mandibular third molar was removed one month ago, but its socket was still open. A panoramic radiograph revealed an ill-fitting root canal treatment of the right mandibular second premolar with periapical radiolucency. The same image was found around the left mandibular third molar which presented a coronal crack (Figure 1).

**Figure 2:**
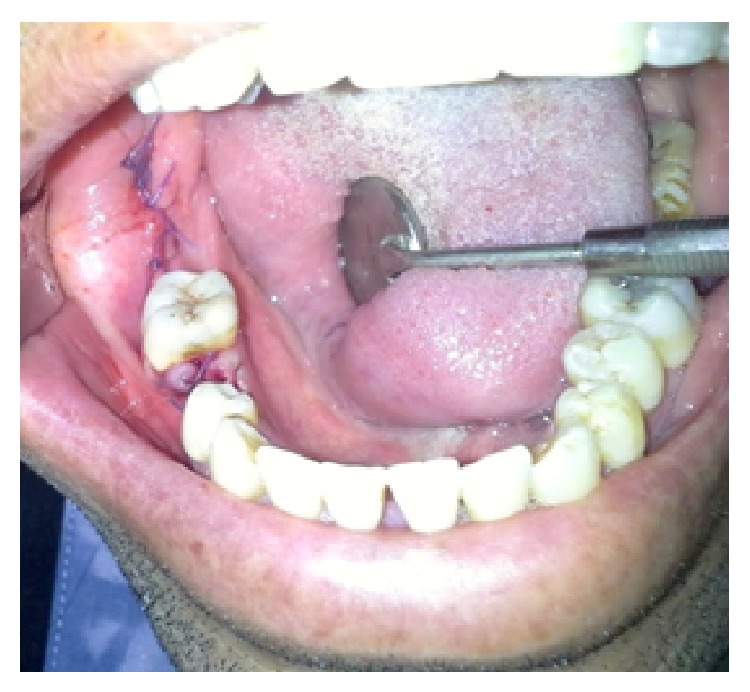
Intraoperative view after extraction of the mandibular right second premolar and debridement of the mandibular right third molar socket.

Supragingival scaling and extraction of the left maxillary second molar were performed first (Figure 2).

**Figure 3:**
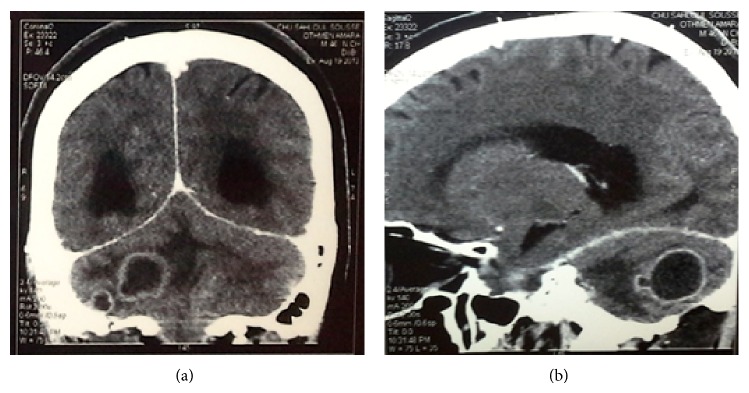
Computed tomography scan: coronal (a) and sagittal (b) views showing 3 cerebellar ring-enhancing mass lesions.

The patient, still under antibiotic treatment (metronidazole, vancomycin, and cefotaxime), exhibiting a slight improvement in walking and presenting no sign of headache nor vomiting, was permitted to be discharged on the ninth postoperative day and to continue the dental care, although it was not ended yet as mentioned in the treatment plan, by his general practitioner. Four days later, he was readmitted to the neurosurgery service owing to the new onset of walking disorders and severe headaches. Moreover, the patient complained of right arm and leg weakness. A new cerebral scan was ordered and it showed recurrence of the cerebellar abscesses that compressed the brain stem and the fourth ventricle with a sign of an active biventricular hydrocephalus (Figures 3(a) and 3(b)).

**Figure 4:**
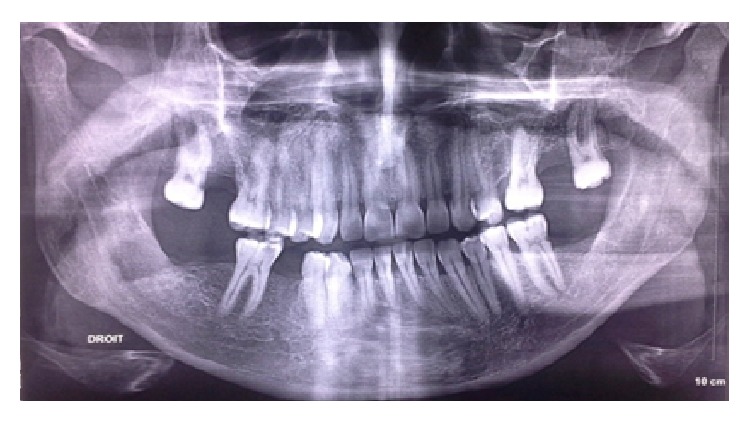
A 3-month postoperative panoramic radiograph.

The patient underwent a large craniectomy and the infectious diseases service recommended maintaining him under metronidazole, vancomycin, and cefotaxime as described previously. He was readdressed to the dentistry service in order to achieve the required dental care: the left mandibular third molar and the right mandibular second premolar were extracted and curettage and local debridement of the socket of the right mandibular third molar were performed (Figure 4). At the end, cefotaxime was substituted by ciprofloxacin because it caused an allergic reaction marked by multiple erythematous papules on the patient's neck. He was discharged on the twenty-ninth postoperative day, and by that time he had experienced a full return of his right arm strength, with some residual weakness in his right leg.

## 3. Discussion

Brain abscesses are still an important cause of mortality and morbidity despite the improvement in diagnosis and treatment modalities in recent decades [3, 7]. They can result from dental or maxillofacial infections constituting direct threats to the patient's life [1]. But they usually develop from a contiguous focus of infection, most often from infections in the middle ear, mastoid cells, or paranasal sinuses [3]. In fact, otogenic brain abscess may constitute about 70% of brain abscess [8]. Middle ear suppurative disease may extend to temporal lobe or cerebellum via various routes. They are more dangerous than sinogenic abscesses (frontal and parietal) and often more resistant to antibiotics. Some authors reported that otogenic abscesses have worse outcome than others.

The most common sites of cerebral abscesses are the temporal lobes (42%) and the cerebellum [6]. They may occur following cranial trauma, or craniomaxillofacial surgery, or secondary to a septic focus elsewhere and spread either by direct extension or by haematological route [1], affecting males two to three times more than females, in the sixth decade [9]. This is not in accordance with our case as the patient was much younger. Age and immunity disorders are risk factors for the development of brain abscesses [10].

The oral cavity is considered as being home to a rich and abundant microflora. Actually, dental plaque contains one of the most concentrated accumulations of microorganisms in the human body. To be more specific, approximately 350 different bacterial strains can be isolated in marginal periodontitis and 150 in endodontic infections [1].

Brain abscesses are frequently polymicrobial, with the most commonly isolated microorganisms being* Streptococcus* species, followed by* Staphylococcus* species and* Proteus* species [6, 8, 11–14]. In recent years, anaerobic microorganisms are being more frequently isolated from brain abscess. The most common anaerobic bacteria are* Bacteroides* (especially* B. fragilis*),* Fusobacterium*, and anaerobic* Streptococcus* types and their usually mixed growth is seen in culture [14, 15]. Nevertheless, several studies reported that no microorganism had been isolated and the most important factor responsible for the sterile culture is the usage of antibiotics before surgical intervention [8, 11, 16, 17].

Clinic symptoms and signs are nonspecific for brain abscesses; they show difference depending on the size and location, the virulence of infecting organisms, and underlying systemic conditions [3, 18–20].

The most common symptoms are headache, nausea, vomiting, fever, focal neurological deficits, and alteration of mental status [3, 7, 17, 19, 20]. Our patient presented several of these symptoms.

Routine laboratory tests are not helpful in diagnosis. According to the study of Hakan et al. [21], there is a significant correlation between leukocytosis (above 20.000) with poor outcome and high fever (>38.5°C) with mortality.

For Muzumdar et al. [22], there are no pragmatic rules for treatment of brain abscess and each case must be individualized and treated on its own merits. The mainstay of the treatment includes prompt action and institution of antibiotic therapy. Penicillin and chloramphenicol have long been used until we have opted for cefotaxime/ceftriaxone/ceftazidime, vancomycin, and metronidazole, and this is in agreement with the treatment administered to the patient.

Steroid administration should be generally avoided unless the patient demonstrates signs of meningitis or disproportionate cytotoxic edema posing a life threatening problem.

Legg advocated anticonvulsant therapy for 5 years to all patients with cerebral abscess. Discontinuation of antiepileptic drugs can be considered when patient is seizure-free for at least 2 years after surgery and electroencephalogram [23].

## 4. Conclusion

A cerebral abscess linked to a dental source is a rare occurrence and its clinical features are often nonspecific; thus the diagnosis may be delayed. Most patients symptoms are headache, nausea, and vomiting. However, fever and neurological signs are notably absent in the early stages [6]. Once the diagnosis has been made, treatment consists of 3 components: administration of large spectrum antibiotics that should be adjusted when the microorganism and the pattern of resistance are known, pus drainage, and eradication of the primary focus of infection [6]. It is worth noting that this latter component calls upon dental clinicians to pay more attention in the identification and treatment of oral and odontogenic sources for brain abscesses [24].
